# Correlation between acylcarnitine/free carnitine ratio and cardiopulmonary exercise test parameters in patients with incident dialysis

**DOI:** 10.3389/fphys.2023.1155281

**Published:** 2023-03-07

**Authors:** Wataru Ito, Kiyotaka Uchiyama, Ryunosuke Mitsuno, Erina Sugita, Takashin Nakayama, Toshinobu Ryuzaki, Rina Takahashi, Yoshinori Katsumata, Kaori Hayashi, Takeshi Kanda, Naoki Washida, Kazuki Sato, Hiroshi Itoh

**Affiliations:** ^1^ Department of Endocrinology, Metabolism and Nephrology, Keio University School of Medicine, Tokyo, Japan; ^2^ Department of Cardiology, Keio University School of Medicine, Tokyo, Japan; ^3^ Institute for Integrated Sports Medicine, Keio University School of Medicine, Tokyo, Japan; ^4^ Department of Nephrology, International University of Health and Welfare Narita Hospital, Narita, Chiba, Japan

**Keywords:** carnitine, cardiopulmonary exercise test, dialysis, peak oxygen consumption, VE/VCO_2_ slope, work rate, chronotropic index

## Abstract

**Objective:** Diminished physical capacity is common and progressive in patients undergoing dialysis, who are also prone to deficiency in carnitine, which plays a pivotal role in maintaining skeletal muscle and cardiac function. The present study aimed to evaluate the association of carnitine profile with exercise parameters in patients with incident dialysis.

**Design and Methods:** This was a single-center cross-sectional study including 87 consecutive patients aged 20–90 years who were initiated on dialysis in Keio University Hospital between December 2019 and December 2022 and fulfilled the eligibility criteria. Exercise parameters were evaluated *via* cardiopulmonary testing (CPX) using the electronically braked STRENGTH ERGO 8 ergometer, whereas the carnitine profile was assessed by determining serum free carnitine (FC), acylcarnitine (AC) levels and AC/FC ratio.

**Results:** The mean cohort age was 62.1 ± 15.2 years, with male and hemodialysis predominance (70% and 73%, respectively). AC/FC was 0.46 ± 0.15, and CPX revealed peak oxygen consumption (VO_2_) of 13.9 ± 3.7 (mL/kg/min) with percent-predicted peak VO_2_ of 53.6% ± 14.7% and minute ventilation (VE)/carbon dioxide output (VCO_2_) slope of 35.1 ± 8.0. Fully-adjusted multivariate linear regression analysis showed that AC/FC was significantly associated with decreased peak VO_2_ (β, −5.43 [95% confidence interval (CI), −10.15 to −0.70]) and percent-predicted peak VO_2_ (β, −19.98 [95% CI, −38.43 to −1.52]) and with increased VE/VCO_2_ slope (β, 13.76 [95% CI, 3.78–23.75]); FC and AC did not exhibit similar associations with these parameters. Moreover, only AC/FC was associated with a decreased peak work rate (WR), percent-predicted WR, anaerobic threshold, delta VO_2_/delta WR, and chronotropic index.

**Conclusion:** In patients on incident dialysis, exercise parameters, including those related to both skeletal muscle and cardiac function, were strongly associated with AC/FC, a marker of carnitine deficiency indicating altered fatty acid metabolism. Further studies are warranted to determine whether carnitine supplementation can improve exercise capacity in patients on incident dialysis.

## 1 Introduction

Diminished physical capacity, which is assessed using peak oxygen consumption (VO_2_), muscle strength, and other exercise tests, is common and progressive in patients with chronic kidney disease (CKD), especially in those with end-stage renal disease (ESRD) undergoing dialysis (Fahal, 2014). Additionally, reduced peak VO_2_ and muscle weakness accompanying CKD are associated with high risk of morbidity and mortality, although the etiology is multifactorial and remains unclear (Tentori et al., 2010; Roshanravan et al., 2013; Pereira et al., 2015). Therefore, there is an urgent need to elucidate the mechanisms underlying reduced exercise capacity and to develop interventions that can improve exercise capacity in patients with CKD.

Carnitine, which is mostly distributed in muscle cells, promotes adenosine triphosphate production through fatty acid β-oxidation by transporting long-chain fatty acids from the cytoplasm to the mitochondria (Evans and Fornasini, 2003). Therefore, carnitine plays a pivotal role in maintaining skeletal muscle and cardiac function. Patients with CKD, especially those on dialysis, are prone to carnitine deficiency, which is defined as a decrease in blood levels of free carnitine (FC) or an increase in acylcarnitine/FC ratio (AC/FC), due to decreased renal carnitine production, decreased dietary intake, and removal of carnitine during dialysis (Hiatt et al., 1992). A high AC/FC ratio may be owing to a relative deficiency of FC, which is required to eliminate toxic acyl CoA levels in the mitochondria and it has been associated with various metabolic abnormalities (McCann et al., 2021). Consequently, a high AC/FC may be correlated with mitochondrial dysfunction and reduced exercise capacity (Gnoni et al., 2020; McCann et al., 2021). Indeed, carnitine supplementation in patients on hemodialysis can improve muscle symptoms and cardiac function parameters, such as left ventricular ejection fraction (EF), and may prevent intradialytic hypotension (Eknoyan et al., 2003; Higuchi et al., 2016). However, the association between exercise capacity and carnitine profile is not well established.

A renal rehabilitation clinic, which was opened in Keio University Hospital in December 2019, primarily provides cardiopulmonary exercise testing (CPX) and individualized exercise prescriptions to patients on incident dialysis. CPX is the gold-standard method used to independently assess muscle, cardiac, and pulmonary function during exercise (Adachi, 2017). In the present study, we explored the exercise capacity assessed as CPX in incident dialysis patients and used FC, AC, and AC/FC among carnitine profile to explore the correlation with clinical CPX parameters, including peak VO_2_ and minute ventilation (VE)/carbon dioxide output (VCO_2_) slope, which are well established prognostic markers for serious cardiovascular events (Poggio et al., 2010). This information may help to clarify the role of carnitine deficiency in the reduced exercise capacity of patients undergoing dialysis, especially its effect on skeletal muscle and cardiac function, both of which are major determinants of physical capacity as independently determined by CPX. Furthermore, a better understanding of the correlation between exercise capacity and carnitine could help in the development of therapeutic approaches based on carnitine supplementation to alleviate the detrimental effects of reduced physical performance in dialysis patients.

## 2 Methods

### 2.1 Study population

Consecutive patients aged 20–90 years who were newly initiated on hemodialysis or peritoneal dialysis (PD) in Keio University Hospital between December 2019 and December 2022 were assessed for their eligibility to undergo CPX after several dialysis sessions; CPX was therefore usually performed within 1 month following dialysis induction. Patients with the following conditions were excluded from the study (Uchiyama et al., 2021): uncontrolled hypertension (blood pressure > 180/110 mmHg); severe anemia (hemoglobin <7 mg/dL); active proliferative diabetic retinopathy; current heart failure (HF) (New York Heart Association class IV); symptomatic coronary artery disease, aortic disease, or cerebrovascular disease within 3 months; symptomatic fatal arrhythmia; significant valvular heart disease; active infections including Coronavirus Disease 2019; marked cognitive dysfunction, intellectual disability, or mental disorders preventing the patient from following instructions; and difficulty in walking without aid due to orthopedic issues, peripheral artery disease, or cerebrovascular disease. Additionally, patients who weighed more than 130 kg were excluded because the load capacity of the ergometer used in the study (STRENGTH ERGO 8, Mitsubishi Electric Engineering Company, Japan) was limited to 130 kg.

### 2.2 Study design

This was a single-center cross-sectional observational study. The study protocol was reviewed and approved by the Ethics Committee of Keio University Hospital (approval no: 20221168. The study adhered to the principles of the Declaration of Helsinki. Written informed consent to undergo CPX and participate in the other study (approval no: 2014023) was obtained from all participants, and informed consent for participation in the present study and for publication was obtained using an online opt-out form.

### 2.3 CPX

In patients undergoing hemodialysis, CPX was performed on a day without hemodialysis between regularly scheduled hemodialysis sessions. In patients undergoing PD, CPX was performed at any time as long as the patient’s abdomen was free of dialysis fluid. Additionally, CPX was performed after several dialysis sessions following the confirmation of absence of ESRD symptoms, such as uremia and congestive HF. On the day of CPX, the patients were instructed to avoid heavy physical activity before testing. Height (m) and body weight (kg) measured immediately before testing were used to calculate body mass index (BMI).

As previously described, CPX was performed using the electronically braked STRENGTH ERGO 8 ergometer (Shiraishi et al., 2018; Seki et al., 2021). This device is driven by a servo motor that can be easily programmed with different exercise programs by a personal computer and enables a power range from less than 50 W (including 0 W) to 600 W, with adjustable increments of 1 W. Briefly, following a 2-min rest, the patient performed warm-up pedaling at 0 W for 2 min, followed by exercising with a progressively increasing intensity until volitional exhaustion, defined as the inability to no longer maintain the current pedaling rate. Intensity was increased by 10–20 W/min, defined as the RAMP protocol. The pedaling frequency was set at 60 revolutions/min. Once the exercise test was terminated, the patients were instructed to stop pedaling and to stay on the ergometer for 3 min.

Heart rate (HR) was continuously monitored and blood pressure was measured every minute during CPX. Peak work rate (WR) and peak HR were recorded during CPX, and chronotropic index (%) was evaluated using the following equation: chronotropic index (%) = (peak HR − rest HR)/(220 − age − rest HR) × 100 (Usui et al., 2021).

### 2.4 Respiratory gas analysis

Expired gas parameters were measured and analyzed using a breath-by-breath automated system (Vmax; Nihon Koden, Tokyo, Japan), as previously described (Shiraishi et al., 2018; Seki et al., 2021). Briefly, respiratory gas exchange parameters, including VE, VO_2_, and VCO_2_, were continuously monitored and measured using 10-sec averages. Peak VO_2_ was defined as mean oxygen consumption during the last 30 s of exercise. The VE/VCO_2_ slope was based on data from the onset of exercise to the respiratory compensation point and was calculated using linear regression analysis of data acquired throughout the entire exercise period. Anaerobic threshold (AT) was determined using V-slope and time trend of VE/VO_2_, VE/VCO_2_, gas exchange ratio (R; VCO_2_/VO_2_), and end-tidal O_2_ and CO_2_, as previously described (Itoh et al., 2013). AT was expressed as VO_2_ and divided by body weight. Ratio of VO_2_ increase (delta VO_2_) to WR increase (delta WR) was determined using linear regression of VO_2_ plots (Itoh et al., 1992).

### 2.5 Data collection

Demographic data, including age, sex, dialysis modality (hemodialysis or PD), presence or absence of diabetic kidney disease (DKD), history of cerebro-cardiovascular disease (CCVD), and use of antihypertensive drugs, were obtained from the medical records. Smoking status was recorded as non-smoker or smoker, which was further categorized as current smoker or ex-smoker.

For biochemical analyses, blood samples were collected immediately before hemodialysis during the mid-week session in patients undergoing hemodialysis; blood samples were collected at any time in patients undergoing PD. Serum carnitine profile was determined using an enzyme cycling method, and carnitine deficiency was defined as decreased serum FC level and increased AC/FC (Uchiyama et al., 2021). The following serum parameters were also evaluated: albumin, hemoglobin, urea nitrogen, creatinine, estimated glomerular filtration rate (eGFR) calculated using serum creatinine level with the three-variable Japanese equation (Matsuo et al., 2009), sodium, potassium, chloride, corrected calcium, phosphate, and magnesium. Plasma levels of brain natriuretic peptide (BNP) were also measured to assess fluid status. Moreover, percent left ventricular EF, ratio of early diastolic filling velocity to early mitral lateral annulus velocity, and left ventricular mass index (LVMI) were measured using echocardiography.

### 2.6 Outcome measures

Primary outcomes were peak VO_2_ and VE/VCO_2_ slope, and secondary outcomes were peak WR, AT, delta VO_2_/delta WR, and chronotropic index; all were determined during CPX. Additionally, given that peak VO_2_ and peak WR are affected by age, sex, and body weight, percent-predicted peak VO_2_ (%peak VO_2_) and percent-predicted peak WR (%peak WR) were obtained using age-, sex-, and body weight-predicted values in healthy Japanese subjects for assessment (Itoh et al., 2013). Correlation of FC level and AC/FC with these exercise parameters were analyzed using regression analyses.

### 2.7 Statistical analysis

Normality of continuous data was tested using the Kolmogorov–Smirnov test. Normally and non-normally distributed variables were presented as means ± standard deviation and medians (25th–75th percentile), respectively. Skewed data were log-transformed before analyses. Categorical data were expressed as numbers (%).

Statistical analyses included simple linear regression analysis followed by multiple linear regression analysis, with exercise parameters included as dependent variables. Results were reported using β coefficients (95% confidence intervals [CIs]). For simple linear regression analysis, only FC, AC, and AC/FC ratio were included as independent variables. Unpaired Student’s *t*-test was used for comparisons between the following prespecified patient subgroups: male versus female, DKD versus non-DKD, hemodialysis versus PD, presence versus absence of history of CCVD, presence versus absence of smoking, presence versus absence of β-blocker use.

Multivariate regression models were created based on variables that were previously reported as predictors of exercise capacity and were not dependent on an association with exercise capacity identified in simple linear regression analysis in the current study (Sietsema et al., 2002; Zuo et al., 2013; Chinnappa et al., 2021). Consequently, age, sex, BMI, DKD, serum albumin, hemoglobin, eGFR, BNP or EF (considered as markers of HF), and FC, AC or AC/FC (considered as markers of carnitine deficiency), were selected as independent variables. Due to the multicollinearity between the BNP level and EF, these variables were not included in the same models. Moreover, having into account the multicollinearity among FC, AC levels, and AC/FC ratio, these variables were not included either in the same models. In addition, according to the “15 subjects per variable” rule to prevent overfitting and considering the limited study population of approximately 90 patients, six independent variables were selected in Model 1 and the number of independent variables inflated stepwise from Model 1 to Model 2. Consequently, the analyses were performed using two distinct models as follows. Model 1 was a minimally-adjusted model with age, sex, log-transformed BMI, hemoglobin level, cardiac marker (EF in Model 1A or log-transformed BNP level in Model 1B), and carnitine profile (FC level, AC level or AC/FC ratio). Model 2 was the fully-adjusted model further adjusted for serum albumin level, eGFR, and DKD. However, peak VO_2_ and AT were already divided by body weight; therefore, BMI was excluded from independent variables when peak VO_2_, %peak VO_2_, and AT were selected as dependent variables. Additionally, considering that β-blockers slow HR, β-blocker use was included as an independent variable when chronotropic index was set as a dependent variable.

All statistical analyses were performed using EZR (Saitama Medical Center, Jichi Medical University, Saitama, Japan), a graphical user interface for R (The R Foundation for Statistical Computing, Vienna, Austria) (Kanda, 2013). A two-sided *p*-value of < 0.05 was considered to indicate statistical significance.

## 3 Results

### 3.1 Clinical characteristics

**FIGURE 1:**
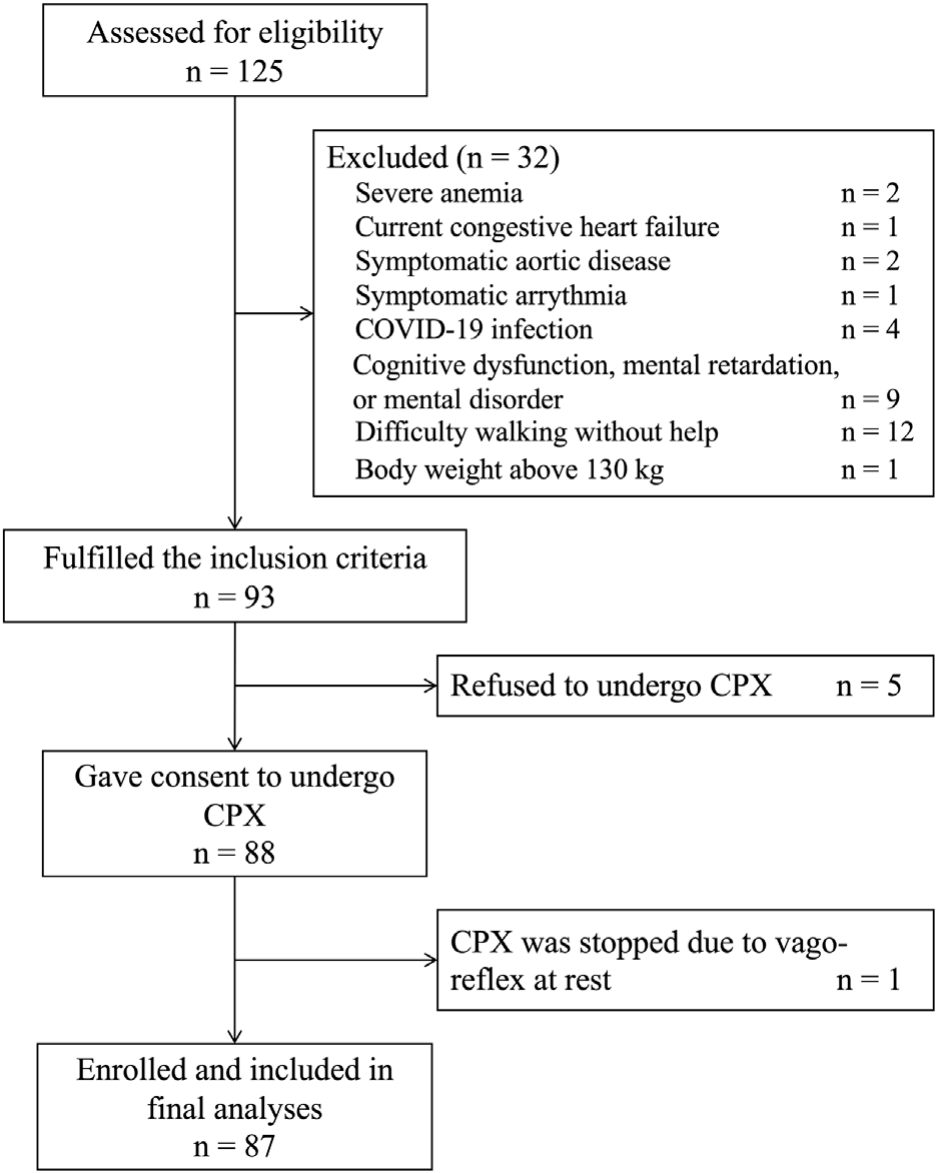
Flow chart of the study participants. COVID-19, Coronavirus Disease 2019; CPX, cardiopulmonary testing.

Among 125 Japanese patients who were initiated on dialysis during the study period, 87 patients who fulfilled the inclusion and exclusion criteria were included in final analyses (Figure 1). The clinical characteristics of the study cohort are summarized in Table 1. Briefly, mean age was 62.1 ± 15.2 years, 70% of the patients were male, and 73% of the patients were on hemodialysis. In the entire cohort, the mean FC level was 42.0 ± 11.9 μmol/L and the mean AC/FC was 0.46 ± 0.15. Of note, an AC/FC > 0.40 indicates altered fatty acid metabolism (Schreiber, 2006).

**TABLE 1:** Background characteristics of the study population.

Variables	All (n = 87)
Age (year)	62.1 ± 15.2
Sex (% male)	61 (70%)
Diabetic kidney disease	26 (30%)
Hemodialysis/peritoneal dialysis	73/14 (84/16%)
Cerebro-cardiovascular disease	23 (26%)
Smoker	46 (53%)
β blocker use	24 (28%)
Height (cm)	164.5 ± 7.2
Body weight (kg)	62.7 ± 16.3
Body mass index (kg/m^2^)	22.2 (20.1–25.3)
Resting heart rate (beats/min)	78.8 ± 14.9
Albumin (g/L)	3.3 ± 0.5
Hemoglobin (g/dL)	10.1 ± 1.3
Urea nitrogen (mg/dL)	54.9 ± 14.8
Creatinine (mg/dL)	8.2 ± 2.4
eGFR (mL/min/1.73 m^2^)	5.9 ± 1.7
Sodium (mEq/L)	137.5 ± 3.1
Potassium (mEq/L)	4.2 ± 0.6
Chloride (mEq/L)	102.3 ± 4.3
Corrected calcium (mg/dL)	8.9 ± 0.6
Phosphorus (mg/dL)	5.0 ± 1.2
Magnesium (mg/dL) (*n* = 83)	2.2 ± 0.4
Brain natriuretic peptide (pg/mL)	171.1 (72.4–431.2)
Free carnitine (μmol/L)	42.0 ± 11.9
Acylcarnitine (μmol/L)	19.1 ± 7.7
AC/FC	0.46 ± 0.15
Echocardiography	
LVEF (2D) (%)	62.8 ± 11.0
LVMI (g/m^2^)	113.3 ± 30.7
E/e' (sep) (*n* = 86)	13.2 ± 4.2
Exercise parameters	
Peak VO_2_ (mL/kg/min)	13.9 ± 3.7
%peak VO_2_ (%)	53.6 ± 14.7
Peak work rate (kg)	61.8 ± 22.8
%peak work rate (%)	54.3 ± 15.7
VE/VCO_2_ slope	35.1 ± 8.0
Delta VO_2_/delta WR (mL/min/W)	7.8 ± 2.0
Gas exchange ratio	1.14 ± 0.11
Peak heart rate (bpm)	120.1 ± 24.0
Heart rate reserve (%)	48.1 ± 22.0

Abbreviations: eGFR, estimated glomerular filtration rate; VO_2_, oxygen consumption; VE, minute ventilation; VCO_2_, carbon dioxide output; AC/FC, acylcarnitine/free carnitine ratio; LVEF (2D), left ventricular ejection fraction (2-dimensional); LVMI, left ventricular mass index; and E/e’ (sep), ratio of early diastolic filling velocity to early mitral lateral annulus velocity (septum).

CPX was performed a median of 7 (5–21) days after dialysis induction. The mean peak VO_2_ and peak WR were 13.9 ± 3.7 mL/kg/min and 61.8 ± 22.8 W, respectively. Both parameters were significantly lower than the estimated values in healthy Japanese individuals, and %peak VO_2_ and %peak WR were 53.6% ± 14.7% and 54.3% ± 15.7%, respectively. Chronotropic index was also lower than its estimated value in healthy individuals, with a chronotropic index of 48.1 ± 22.0. The VE/VCO_2_ slope was 35.1 ± 8.0, which was higher than the cutoff value of 29.9, implying a negligible risk of major cardiac events (Arena et al., 2008).

Comparison of the exercise parameters between specific patient subgroups revealed that %peak VO_2_ and peak WR were significantly higher in male patients than in female patients (*p* < 0.001 and *p* = 0.003, respectively) (Table 2). DKD was associated with lower %peak WR, AT, and chronotropic index and with higher delta VO_2_/delta WR (*p* < 0.05, *p* = 0.04, *p* = 0.001, and *p* = 0.04, respectively) (Table 3). Additionally, history of CCVD and smoking were associated with higher VE/VCO_2_ slope (*p* = 0.03 and *p* = 0.04, respectively).

**TABLE 2:** Comparisons of primary outcomes between subgroups.

Variables	Peak VO_2_	*p*-value	%Peak VO_2_	*p*-value	VE/VCO_2_ slope	*p*-value
Sex		0.27		<0.001		0.95
Male	14.2 ± 3.7		58.3 ± 13.8		35.1 ± 9.9	
Female	13.2 ± 3.8		42.6 ± 10.4		35.2 ± 7.1	
DKD		0.25		0.49		0.81
Yes	13.2 ± 3.3		52.0 ± 15.9		35.5 ± 8.0	
No	14.2 ± 3.8		54.3 ± 14.2		35.0 ± 8.0	
Modality		0.56		0.99		0.82
HD	13.8 ± 3.8		53.6 ± 14.4		35.2 ± 8.2	
PD	14.4 ± 3.3		53.7 ± 16.8		34.7 ± 6.8	
CCVD		0.1		0.6		0.03
Yes	12.8 ± 4.1		54.1 ± 15.9		38.3 ± 8.4	
No	14.3 ± 4.1		52.3 ± 10.8		34.0 ± 7.6	
Smoking		0.45		0.3		0.04
Yes	13.6 ± 3.7		55.2 ± 15.1		36.8 ± 9.1	
No	14.2 ± 3.7		51.9 ± 14.2		33.3 ± 6.2	
β-blocker		0.62		0.6		0.83
Yes	13.6 ± 2.3		52.3 ± 10.1		35.4 ± 6.0	
No	14.0 ± 4.1		54.1 ± 16.2		35.0 ± 8.7	

Abbreviations: VO_2_, oxygen consumption; VE, minute ventilation; VCO_2_, carbon dioxide output; DKD, diabetic kidney disease; HD, hemodialysis; PD, peritoneal dialysis; CCVD, cerebro-cardiovascular disease.

**TABLE 3:** Comparisons of secondary outcomes between subgroups.

Variables	Peak WR	*p*-value	%Peak WR	*p*-value	AT	*p*-value	Delta VO_2_/delta WR	*p*-value	Chronotropic index	*p*-value
Sex		0.002		0.1		0.61		0.21		0.49
Male	66.5 ± 22.1		56.1 ± 15.6		9.4 ± 2.1		8.0 ± 2.1		47.0 ± 21.2	
Female	50.7 ± 20.7		50.1 ± 15.6		9.7 ± 2.2		7.4 ± 1.6		50.6 ± 24.1	
DKD		0.56		<0.05		0.04		0.04		0.001
Yes	64.0 ± 23.7		49.2 ± 18.5		8.8 ± 1.7		8.5 ± 1.8		36.8 ± 20.6	
No	60.9 ± 22.5		56.5 ± 14.0		9.8 ± 2.2		7.6 ± 2.0		52.9 ± 20.9	
Modality		0.78		0.32		0.48		0.42		0.18
HD	61.5 ± 23.4		53.6 ± 15.9		9.4 ± 2.0		7.9 ± 2.0		46.7 ± 21.8	
PD	63.4 ± 20.0		58.2 ± 14.9		9.9 ± 2.5		7.5 ± 1.5		55.3 ± 22.5	
CCVD		0.2		0.53		0.31		0.4		0.05
Yes	56.6 ± 22.4		52.5 ± 15.3		9.1 ± 2.4		7.5 ± 2.2		40.5 ± 19.7	
No	63.7 ± 22.8		55.0 ± 16.0		9.6 ± 2.4		8.0 ± 1.9		50.8 ± 22.3	
Smoking		0.49		0.67		0.13		0.51		0.07
Yes	63.4 ± 22.2		55.0 ± 15.5		9.2 ± 2.1		7.7 ± 1.9		44.1 ± 20.5	
No	60.0 ± 23.5		53.6 ± 16.2		9.8 ± 2.1		8.0 ± 2.1		52.5 ± 23.1	
β-blocker		0.59		0.58		0.13		0.21		0.11
Yes	64.0 ± 21.0		52.8 ± 11.8		8.9 ± 1.5		8.3 ± 1.7		42.0 ± 20.2	
No	61.0 ± 23.5		54.9 ± 17.0		9.7 ± 2.3		7.7 ± 2.0		50.4 ± 22.4	

Abbreviations: WR, work rate; AT, anaerobic threshold; VO_2_, oxygen consumption; DKD, diabetic kidney disease; HD, hemodialysis; PD, peritoneal dialysis; CCVD, cerebro-cardiovascular disease.

### 3.2 Association of the carnitine profile with primary outcomes

The results of simple linear regression analyses are shown in Table 4. Briefly, AC/FC was significantly correlated with peak VO_2_ (β, −7.18 [95% CI, −12.11 to −2.24]), %peak VO_2_ (β, −30.23 [95% CI, −49.65 to −10.82]), and VE/VCO_2_ slope (β, 14.8 [95% CI, 4.14–25.47]), whereas FC level did not correlate with any of these variables (*p* = 0.27, *p* = 0.29, and *p* = 0.15, respectively). The AC level did not show any significant correlation with either of these variables (*p* = 0.18, *p* = 0.14, and *p* = 0.27, respectively).

**TABLE 4:** Simple linear regression analysis to determine the association of clinical parameters with primary outcomes.

Variables	Peak VO_2_		%Peak VO_2_		VE/VCO_2_ slope	
	β (95% CI)	*p*-value	β (95% CI)	*p*-value	β (95% CI)	*p*-value
Free carnitine (μmol/L)	0.04 (−0.03–0.10)	0.27	0.14 (−0.12–0.40)	0.29	−0.11 (−0.24–0.04)	0.15
Acylcarnitine (μmol/L)	−0.69(-0.17–0.03)	0.18	−0.30(−0.71–0.10)	0.14	0.12(−0.10–0.35)	0.27
AC/FC	−7.18 (−12.11 to −2.24)	0.005	−30.23 (−49.65 to −10.82)	0.003	14.8 (4.14–25.47)	0.007

Abbreviations: VO_2_, oxygen consumption; VE, minute ventilation; VCO_2_, carbon dioxide output; 95% CI, 95% confidence interval; AC/FC, acylcarnitine/free carnitine ratio.

In multiple linear regression analyses, AC/FC remained a significant predictor of peak VO_2_, %peak VO_2_, and VE/VCO_2_ slope in all models whereas FC level was not a significant predictor of any of these parameters (Table 5). Specifically, in the fully-adjusted Model 2B, AC/FC was significantly and negatively correlated with peak VO_2_ (β, −5.43 [95% CI, −10.15 to −0.70]) and %peak VO_2_ (β, −19.98 [95% CI, −38.43 to −1.52]) and was significantly and positively correlated with VE/VCO_2_ slope (β, 13.76 [95% CI, 3.78–23.75]). FC and AC levels did not show any significant correlation with these primary outcomes.

**TABLE 5:** Simple linear regression analysis to determine the association of clinical parameters with secondary outcomes.

Variables	Peak WR		%Peak WR		AT		Delta VO_2_/delta WR		Chronotropic index	
	β (95% CI)	*p*-value	β (95% CI)	*p*-value	β (95% CI)	*p*-value	β (95% CI)	*p*-value	β (95% CI)	*p*-value
Free carnitine (μmol/L)	0.24 (−0.17–0.65)	0.24	0.22 (−0.06–0.50)	0.11	0.02 (−0.02–0.06)	0.26	0.01 (−0.03–0.05)	0.57	0.06 (−0.34–0.45)	0.78
Acylcarnitine (μmol/L)	−0.17(−0.81–0.46)	0.59	−0.26(−0.70–0.17)	0.23	−0.03(−0.08–0.03)	0.38	−0.03(−0.08–0.03)	0.33	−0.39(−1.00–0.22)	0.21
AC/FC	−34.03 (−64.91 to −3.15)	0.03	−36.78 (−57.22 to −16.34)	<0.001	−7.18 (−12.11 to −2.24)	0.005	−3.09 (−5.94 to −0.23)	0.03	−32.43 (−62.33 to −2.54)	0.03

Abbreviations: WR, work rate; AT, anaerobic threshold; VO_2_, oxygen consumption; 95% CI, 95% confidence interval; AC/FC, acylcarnitine/free carnitine ratio.

### 3.3 Association of the carnitine profile with secondary outcomes

The results of simple linear regression analyses for secondary outcomes are shown in Table 6. Briefly, AC/FC was significantly negatively correlated with peak WR (β, −34.03 [95% CI, −64.91 to −3.15]), %peak WR (β, −36.78 [95% CI, −57.22 to −16.34]), AT (β, −7.18 [95% CI, −12.11 to −2.24]), delta VO_2_/delta WR (β, −3.09 [95% CI, −5.94 to −0.23]), and chronotropic index (β, −32.43 [95% CI, −62.33 to −2.54]). However, FC level was not significantly correlated with any of these variables (*p* = 0.24, 0.11, 0.26, 0.57, and 0.78, respectively). The AC level was not significantly correlated with either of these variables (*p* = 0.59, 0.23, 0.38, 0.33, and 0.21, respectively).

**TABLE 6:** Multiple linear regression analysis to determine the association of carnitine profile with primary outcomes.

Variables	Peak VO_2_		%Peak VO_2_		VE/VCO_2_ slope	
	β (95% CI)	*p*-value	β (95% CI)	*p*-value	β (95% CI)	*p*-value
Free carnitine (μmol/L)						
Model 1A	0.01 (−0.05–0.07)	0.82	0.05 (−0.18–0.28)	0.66	−0.05 (−0.18–0.08)	0.42
Model 1B	0.002 (−0.06–0.06)	0.94	0.03 (−0.19–0.26)	0.77	−0.04 (−0.16–0.09)	0.53
Model 2A	0.01 (−0.05–0.07)	0.85	0.04 (−0.19–0.28)	0.71	−0.08 (−0.21–0.05)	0.23
Model 2B	0.003 (−0.06–0.06)	0.9	0.04 (−0.20–0.27)	0.76	−0.07 (−0.19–0.06)	0.31
Acylcarnitine (μmol/L)						
Model 1A	−0.08 (−0.17–0.01)	0.07	−0.26 (−0.61–0.09)	0.14	0.14 (−0.06–0.34)	0.17
Model 1B	−0.09 (−0.18–0.001)	0.05	−0.29 (−0.63–0.06)	0.1	0.16 (−0.03–0.35)	0.11
Model 2A	−0.07 (−0.16– 0.02)	0.12	−0.22 (−0.57–0.12)	0.21	0.12 (−0.07–0.32)	0.21
Model 2B	−0.08 (−0.17–0.01)	0.08	−0.26 (−0.61–0.09)	0.14	0.15 (−0.04–0.34)	0.12
AC/FC						
Model 1A	−5.46 (−9.92 to −1.01)	0.02	−20.10 (−37.27 to −2.93)	0.02	11.89 (1.86–21.92)	0.02
Model 1B	−5.37 (−9.82 to −0.91)	0.02	−20.00 (−37.30 to −2.71)	0.01	11.29 (1.51–21.06)	0.02
Model 2A	−5.11 (−9.80 to −0.41)	0.03	−18.52 (−36.81 to −0.24)	<0.05	14.00 (3.46–23.73)	0.009
Model 2B	−5.43 (−10.15 to −0.70)	0.02	−19.98 (−38.43 to −1.52)	0.03	13.76 (3.78–23.75)	0.008

Model 1A is adjusted for age, sex, log-transformed body mass index, hemoglobin, ejection fraction, and free carnitine or AC/FC., Of the variables included in Model 1A, log-transformed brain natriuretic peptide is included instead of ejection fraction in Model 1B. Model 2A and 2B are adjusted for the variables included in Models 1A and 1B, respectively, in addition to serum albumin, estimated glomerular filtration rate, and diabetic kidney disease.

Abbreviations: VO_2_, oxygen consumption; VE, minute ventilation; VCO_2_, carbon dioxide output; 95% CI, 95% confidence interval; AC/FC, acylcarnitine/free carnitine ratio.

In multiple linear regression analyses, AC/FC remained negatively correlated with peak WR, %peak WR, delta VO_2_/delta WR, and chronotropic index, with statistical significance or borderline statistical significance (Table 7). Specifically, in the fully-adjusted Model 2B, AC/FC was significantly and negatively correlated with %peak WR (β, −22.41 [95% CI, −41.66 to −3.16]) and delta VO_2_/delta WR (β, −2.87 [95% CI, −5.66 to −0.08]). AC/FC also tended to negatively correlate with peak WR (β, −24.71 [95% CI, −50.86–1.45]; *p* = 0.06) and chronotropic index (β, −28.74 [95% CI, −58.75–1.28]; *p* = 0.06). AC/FC was significantly and negatively correlated with AT only in Model 1A (β, −2.94 [95% CI, −5.85 to −0.04]). FC and AC levels did not show any significant correlation with these secondary outcomes.

**TABLE 7:** Multiple linear regression analysis determine the association of carnitine profile with secondary outcomes.

Variables	Peak WR		%Peak WR		AT		Delta VO_2_/delta WR		Chronotropic index		
	β (95% CI)	*p*-value	β (95% CI)	*p*-value	β (95% CI)	*p*-value	β (95% CI)	*p*-value		β (95% CI)	*p*-value
Free carnitine (μmol/L)											
Model 1A	0.04 (−0.28–0.37)	0.79	0.14 (−0.10–0.39)	0.24	0.016 (−0.02–0.05)	0.41	0.001 (−0.03–0.04)	0.94		−0.09 (−0.48–0.30)	0.64
Model 1B	0.03 (−0.29–0.34)	0.87	0.12 (−0.11–0.36)	0.3	0.01 (−0.02–0.05)	0.48	0.0002 (−0.03–0.03)	0.99		−0.11 (−0.48–0.26)	0.56
Model 2A	0.07 (−0.26–0.40)	0.68	0.16 (−0.08–0.40)	0.19	0.02 (−0.02–0.05)	0.41	0.007 (−0.03–0.04)	0.69		−0.11 (−0.49–0.27)	0.57
Model 2B	0.06 (−0.27–0.39)	0.73	0.15 (−0.10–0.39)	0.24	0.01 (−0.03–0.45)	0.5	0.01 (−0.03–0.04)	0.73		−0.12 (−0.50–0.25)	0.52
Acylcarnitine (μmol/L)											
Model 1A	−0.26 (−0.76–0.25)	0.31	−0.15 (−0.53–0.24)	0.45	−0.03 (−0.09–0.03)	0.3	−0.04 (−0.09–0.01)	0.13		−0.58 (−1.19–0.03)	0.06
Model 1B	−0.26 (−0.75–0.23)	0.29	−0.17 (−0.53–0.20)	0.36	−0.03 (−0.09–0.02)	0.26	−0.04 (−0.09–0.01)	0.11		−0.55 (−1.12–0.02)	0.06
Model 2A	−0.21 (−0.71–0.29)	0.4	−0.10 (−0.47–0.28)	0.61	−0.02 (−0.08–0.04)	0.47	−0.04 (−0.09–0.01)	0.14		−0.56 (−1.14–0.01)	0.05
Model 2B	−0.23 (−0.72–0.26)	0.36	−0.13 (−0.50–0.23)	0.47	−0.02 (0.08–0.03)	0.39	−0.04 (−0.09–0.01)	0.11		−0.56 (−1.12–0.002)	0.05
AC/FC											
Model 1A	−24.52 (−50.20–1.15)	0.06	−22.97 (−42.25 to −3.70)	0.02	−2.95 (−5.85 to −0.05)	<0.05	−2.64 (−5.32–0.04)	0.05		−32.70 (−63.46 to −1.95)	0.04
Model 1B	−22.60 (−47.79–2.58)	0.08	−21.32 (−39.90 to −2.74)	0.03	−2.83 (−5.71–0.06)	0.05	−2.67 (−5.36–0.02)	0.05		−28.59 (−58.20–1.02)	0.06
Model 2A	−24.97 (−51.30–1.36)	0.06	−22.32 (−41.79 to −2.84)	0.03	−2.30 (−5.32–0.72)	0.13	−3.34 (−6.12 to −0.56)	0.02		−30.39 (−60.88–0.10)	0.05
Model 2B	−24.71 (−50.86–1.45)	0.06	−22.41 (−41.66 to −3.16)	0.02	−2.45 (−5.48–0.57)	0.11	−2.87 (−5.66 to −0.08)	0.04		−28.74 (−58.75–1.28)	0.06

Model 1A is adjusted for age, sex, log-transformed body mass index, hemoglobin, ejection fraction, and free carnitine or AC/FC. Of the variables included in Model 1A, log-transformed brain natriuretic peptide is included instead of ejection fraction in Model 1B. Model 2A and 2B are adjusted for the variables included in Models 1A and 1B, respectively, in addition to serum albumin, estimated glomerular filtration rate, and diabetic kidney disease. Additionally, β blocker use is included for the analyses of chronotropic index.

Abbreviations: WR, work rate; AT, anaerobic threshold; VO_2_, oxygen consumption; 95% CI, 95% confidence interval; AC/FC, acylcarnitine/free carnitine ratio.

## 4 Discussion

The current study, which aimed to determine the association between carnitine deficiency and exercise parameters assessed using CPX, revealed that not a decrease in FC level but an increase in AC/FC was an independent significant predictor of reduced exercise capacity determined using peak VO_2_, %peak VO_2_, and %peak WR. Additionally, an increase in AC/FC was independently associated with impaired cardiac function, including lower VE/VCO_2_ slope and higher delta VO_2_/delta WR.

To our knowledge, this is the first study assessing exercise capacity parameters, including peak VO_2_. Using CPX among patients on incident dialysis. This is also the first study to demonstrate that exercise capacity was severely decreased in these patients, although the participants underwent CPX after several dialysis sessions to improve overt ESRD symptoms. Specifically, the mean peak VO_2_ was 13.9 mL/kg/min in the present study. Patients with HF and a peak VO_2_ of ≤14 mL/kg/min are considered to have poor prognosis (Mancini et al., 1991). The mean peak VO_2_ of the present study population was also significantly lower than that reported in previous studies including patients with CKD: mean peak VO_2_ values of 32.5, 27.3, and 16–20 mL/kg/min were reported in patients with early pre-dialysis CKD and late pre-dialysis CKD and in those on maintenance dialysis, respectively (Painter et al., 2002; Kouidi et al., 2009; Chinnappa et al., 2021). Additionally, peak VO_2_ is affected by age, sex, and body weight; therefore, %peak VO_2_ may be a more reliable indicator of prognosis (Stelken et al., 1996; de Groote et al., 2004). Indeed, %peak VO_2_ was also decreased in the present study (53.6% ± 14.7%). Similarly, in the present study population, %peak WR was severely decreased to 54.3% ± 15.7%.

The VE/VCO_2_ slope is a useful parameter to assess the ventilatory response to exercise and is as effective as peak VO_2_ in the prediction of cardiovascular events and survival in patients with HF. Previous studies have suggested that a VE/VCO_2_ slope of approximately >30–35 is associated with increased mortality, cardiac-related hospitalizations, and serious cardiovascular events (Chua et al., 1997; Gitt et al., 2002; Arena et al., 2008; Poggio et al., 2010; Shen et al., 2015). Although the predictive value of the VE/VCO_2_ slope has not been investigated yet in patients with CKD, the mean VE/VCO_2_ value in the population under study was high (35.1), suggesting that poor ventilation/perfusion matches poor prognosis. The delta VO_2_/delta WR ratio shows the severity of HF and daily living activities in patients with HF. A study including Japanese patients with chronic HF revealed that mortality progressively increased in patients with delta VO_2_/delta WR values of ≥9, 7–9, and <7 mL/min/W (Koike et al., 2002), suggesting that the mean delta VO_2_/delta WR value (7.8) in the present study population was moderately low. Additionally, a decreased chronotropic index may reflect an underlying dysregulation of the autonomic nervous system function. In this regard, it has been reported that a decrease in the chronotropic index to <60% is associated with an increased risk of all-cause mortality, cardiovascular mortality, and hospitalization for HF (Dobre et al., 2013). In the present study, the mean chronotropic index value (48.1%) suggests a severe autonomic nervous system dysfunction in patients on incident dialysis and serves as a reminder that caution is warranted when using the chronotropic index for exercise prescription in these patients.

Carnitine deficiency defined by low FC level or high AC/FC is prevalent in patients on maintenance dialysis, and its prevalence increases in parallel to an increase in dialysis vintage (Schreiber, 2006; Higuchi et al., 2016; Maruyama et al., 2019; Kuwasawa-Iwasaki et al., 2020). The present study was limited to patients with ESRD who recently started dialysis therapy; therefore, decreases in FC levels and increases in AC/FC were not as striking as those reported in previous studies. Interestingly, only AC/FC was an independent and significant predictor of parameters assessed by CPX, including those related to both exercise capacity (e.g., peak VO_2_) and cardiac function (e.g., VE/VCO_2_ slope). A previous study reported that patients with HF showed higher circulating AC levels than control subjects regardless of EF. Moreover, a high AC profile may be significantly associated with high N-terminal pro-BNP levels and serve as an indicator of defective mitochondrial β-oxidation (Zordoky et al., 2015; Ruiz et al., 2017). In addition, the AC/FC ratio was reported to be a predictor of cardiac events in patients with HF and to decrease with the progression of CKD, whereas exercise intervention is able to reduce AC/FC *via* enhancing mitochondrial β-oxidation (Yoshihisa et al., 2017; Uchiyama et al., 2021). Several interventional studies including patients on dialysis evaluated the impact of L-carnitine supplementation to elevate FC levels and to reduce AC/FC (Schreiber, 2006; Kudoh et al., 2013; Higuchi et al., 2016; Maruyama et al., 2019; Kuwasawa-Iwasaki et al., 2020; Yano et al., 2021). The results suggested that L-carnitine supplementation increased lean muscle mass and exercise capacity assessed using leg and handgrip strength, shortened chair stand-up time, and 10-m walk test, while improving cardiac parameters including EF, LVMI, and N-terminal pro-BNP levels. Additionally, it has also been shown that carnitine reduces tissue oxidative damage after exercise and helps the processes of muscle tissue repair and remodeling, while its deficiency has been associated with an increased reactive oxygen species production and fatigue during exercise. Indeed, it has been shown that carnitine supplementation attenuates oxidative stress, enhances antioxidant status, and improves muscle performance, being thus helpful in alleviating fatigue in patients with the end-stage renal disease along with a simultaneous reduction in the AC/FC ratio (Sakurauchi et al., 1998; Fatouros et al., 2010). However, the significant exercise-related effects of L-carnitine supplementation were not consistent across the studies and were not evaluated using parameters measured with CPX, the gold standard of exercise testing. The present study results indicate the importance of carnitine sufficiency, especially the prevention of low AC/FC, in preserving exercise tolerance with cardiac function in patients on incident dialysis. Our findings also support the possibility that carnitine might improve cardiac function parameters reflecting exercise tolerance by lowering AC/FC. Further interventional trials using L-carnitine supplementation are warranted to confirm the beneficial impact of L-carnitine supplementation on CPX parameters.

This observational study has several limitations. First, this was a single-center study with a relatively small sample size, leading to lower power to detect significant determinants. However, the proportion of enrolled patients was very high, as only five patients declined to participate, which minimized the adverse effect of small sample size on external validity. Nonetheless, caution is warranted in extrapolating these results to non-Japanese patients. Second, due to the cross-sectional design, the current study could not determine causality and was limited to demonstrating relationships between variables. Third, the impact of several dialysis sessions on clinical and exercise parameters could not be ruled out, although dialysis modality did not affect any exercise parameters and performing CPX in patients with apparent ESRD-related symptoms including congestive HF and uremia would be unethical. Fourth, the study design did not allow the examination of longitudinal effects of impaired exercise parameters and increased AC/FC on clinical outcomes such as HF-related hospitalization, cardiovascular events, and, more importantly, patient survival. Carefully designed prospective cohort studies and interventional trials are needed to confirm these results. Finally, serum AC and FC might not reflect muscle carnitine levels. Nonetheless, this approach allowed us to explore the correlation between the AC/FC ratio and carnitine since a muscle biopsy to determine its concentration is unethical due to the invasiveness of the procedure.

In conclusion, in this prospective observational cohort study, we demonstrated that increased AC/FC was an independent and significant predictor of deterioration of various exercise parameters determined using the gold-standard CPX, including peak VO_2_, %peak VO_2_, and %peak VO_2_, and of impaired cardiac parameters, including increased VE/VCO_2_ slope and decreased delta VO_2_/delta WR, in Japanese patients on incident dialysis.

## Data Availability

The raw data supporting the conclusion of this article will be made available by the authors, without undue reservation.
